# The Recolonization Concentration Concept: Using Avoidance Assays with Soil Organisms to Predict the Recolonization Potential of Contaminated Sites

**DOI:** 10.3390/toxics10030127

**Published:** 2022-03-05

**Authors:** Mathieu Renaud, Tiago Natal-da-Luz, Rui Ribeiro, José Paulo Sousa

**Affiliations:** CFE—Centre for Functional Ecology, Associated Laboratory TERRA, Department of Life Sciences, University of Coimbra, Calçada Martim de Freitas, 3000-456 Coimbra, Portugal; tiagonluz@iav.uc.pt (T.N.-d.-L.); rui.ribeiro@zoo.uc.pt (R.R.); jps@zoo.uc.pt (J.P.S.)

**Keywords:** copper, behavior, *Eisenia andrei*, *Folsomia candida*

## Abstract

In this study the recolonization concentration concept for soil organisms is presented and validated. This concept is based on the empirically deduced avoidance–recolonization hypothesis, which shows a negative correlation between avoidance (AC_x_) and recolonization (RC_x_) (AC_x_ = RC_100−x_) responses. The concept was validated in a two-step approach composed by (i) individual placement tests, to demonstrate the non-influence of individual placement in a dual chamber avoidance test and (ii) small scale gradient tests to demonstrate that the number of colonizers reaching a soil patch with a certain concentration is independent on their previous exposure to lower concentrations. Overall, data show that avoidance data can be used, when framed under the recolonization concentration concept, to evaluate the recolonization potential of contaminated sites. The recolonization concept is an important theoretical concept that when coupled with spatial modelling tools could be used to tackle the spatial and temporal recovery dynamics of contaminated soil.

## 1. Introduction

Ecological recovery is a key component to consider when establishing acceptable risk levels for specific protection goals and was considered in proposals for the risk assessment of plant protection products [1,2,3,4]. Ecological recovery includes the “community recovery principle” which assumes that the ability of communities to recover allows ecosystems to endure and absorb certain amounts of pollution. As a result, protection goals are allowed to be more permissive because communities are viewed as dynamic systems that can react and recover from stress. In addition to community recovery, risk assessment schemes may also include the species vulnerability concept, an overarching concept which considers not only the species sensitivity to a contaminant, but also its probability of exposure and recovery potential after the occurrence of effects [5].

Recovery at the population level of a previously contaminated site (sensu lato) involves the recolonization, establishment and growth of a population at that site. These processes are dependent on the level of contamination and the existence of a source population either in the edge of the impacted area (external recovery) or from population reservoirs in non-contaminated patches within the impacted area (internal recovery) [6,7]. They are also temporally dependent on the dispersal ability of organisms [8,9], which can be influenced by habitat quality (e.g., food availability) and structure [10,11,12]. The actual knowledge on the recolonization of contaminated sites by soil fauna is relatively scarce. Some studies have shown the effects of different levels of pesticide mixtures on the recolonization of soil microarthropod and macrofauna communities [6,7,13] and the effect of copper on the recolonization of an abandoned arable soil by earthworms [14].

At the ecosystem level or even community level, understanding recovery is a complex task which requires the combined use of experimentation and modelling tools. In fact, an EFSA scientific opinion [4] has highlighted that if community recovery is to be considered in the risk assessment of plant protection products, then population models and specifically spatially and temporally based population models need to be included to tackle population dynamics and their recovery in fields. The use of population models in ecological risk assessment has been widely discussed [15,16,17] and, for in-soil organisms, individual- and matrix-based models have been developed with the collembolan *Folsomia candida* addressing both toxic effects and recolonization issues [18,19,20]. Besides the need to expand these models to other key species of soil fauna from different ecological groups, models must also be improved by including a wider variety of trait data (mainly life history and dispersal traits) obtained by experimentation. For this goal, standard ecotoxicological tests can provide information on toxic effects on several life-history traits (e.g., growth, reproduction). However, for recolonization acquiring experimental data is more difficult since no simple standardized test exists to evaluate the recolonization potential of a soil contaminated with a certain concentration of a chemical. In this paper, we aimed at demonstrating that data from avoidance tests can be used to address the recolonization potential of contaminated soil patches. 

Avoidance tests, such as those performed with the collembolan *F. candida* and the earthworm *Eisenia andrei* [21,22], are a sensitive and cost-effective screening tool to assess toxic effects, especially in site-specific risk assessment [23,24,25]. Avoidance responses towards a certain level of contamination can influence dispersal processes directly, providing important hints about the recolonization process. In fact, some studies have already demonstrated that avoidance behavior is tightly related with the spatial distribution of soil animals in patchily contaminated soils [26] and functions as a behavioral mechanism that allows organisms to reduce their exposure to contaminants [27]. 

A contaminated habitat losing its populations through avoidance will be recolonized as soon as the concentration of the contaminant is sufficiently reduced to become undetected and, thus, unavoided. Therefore, it is expected that the proportion of organisms avoiding a contaminant at a given concentration is negatively correlated with the proportion of organisms unable to detect it and, thus, able to occupy this contaminated habitat. In other words, a contaminant concentration causing a certain percentage of organism avoidance (x, in %) is expected to allow the remaining organisms to colonize this habitat (100−x, in %). This logically deduced prediction—the avoidance–recolonization hypothesis—has its formulation as follows: AC_x_ = RC_100−x_, where AC_x_ (avoidance concentration) is the contaminant concentration eliciting an x% of avoidance and RC (recolonization concentration) is the contaminant concentration allowing a 100−x% of recolonization. In fact, this hypothesis, the avoidance–recolonization hypothesis, has been demonstrated in the aquatic environment with the model species *Daphnia magna* [28].

For this hypothesis to hold true, one assumption needs to be fulfilled: the number of organisms avoiding (when placed in the contaminated soil) and the number of organisms colonizing (when placed in the control soil) a habitat patch presenting a certain concentration of a chemical is not different within an avoidance test. However, in contaminated sites, it is common that gradients of contamination are observed and as such, it is also important for the RC_x_ concept that the number of organisms colonizing a certain soil patch is unaffected by the pattern of previous exposure to lower concentrations those organisms experienced along the gradient. 

In the present study, experiments using two key species of soil fauna (the earthworm *E. andrei* and the collembolan *F. candida*), avoidance behavior as the endpoint and copper as a model substance were performed to empirically validate the avoidance–recolonization hypothesis. To attain this goal, three experimental setups were performed: (1) dual chamber avoidance test to estimate AC_x_ values; (2) dual chamber avoidance tests with the introduction of the test organisms in different areas of the test units to confirm that the final distribution of the test organisms is independent of their initial position; and (3) small-scale gradient tests using test vessels divided into three sections to confirm that the number of colonizers reaching a soil with a certain level of contamination is not influenced by a previous exposure to lower concentrations of the same contaminant. 

## 2. Material and Methods

### 2.1. Test Soil

A sandy-loam natural soil collected from an agricultural field in the suburban limits of the city of Coimbra, Portugal, was used as the test soil. This soil was free of pesticide or fertilizer applications for more than five years. Background copper concentrations were measured in the soil (control samples) for each of the current experiments as described below and averaged 29.4 ± 2.6 mg/kg. The soil was collected from the top 20 cm layer, sieved at 5 mm and defaunated through two freeze–thaw cycles (48 h at −20 °C followed by 48 h at 25 °C). Physical and chemical parameters measured (Table 1) were texture [29], water-holding capacity [30], pH (1 M KCl 1:6 v:v), organic matter content (loss on ignition at 500 °C for 6 h), total N [31] and cation exchange capacity [32].

### 2.2. Test Organisms and Culture Conditions

Earthworms from the species *Eisenia andrei* (Oligochaeta: Lumbricidae) and springtails from the species *Folsomia candida* (Collembola: Isotomidae) were used as test organisms in the experiments. These species are currently used in avoidances tests [23,24] and have standardized ISO guidelines for this type of test [21,22]. Both test species were obtained from laboratory cultures reared under a photoperiod of 16:8 h light:dark at 20 ± 2 °C. The earthworms were kept in plastic containers (36 cm length, 22 cm width, and 11 cm height) using horse manure and *Sphagnum* sp. peat as substrate in a ratio of 1:1 (w:w). Cooked oatmeal was given as food twice a month. Springtails were cultured in cylindrical transparent plastic boxes (11 cm diameter and 4 cm height) using a mixture of plaster of Paris and activated charcoal in a ratio of 11:1 (w:w) as the substrate. Granulated dry yeast was added as food in small amounts to avoid spoilage by fungi. Moldy food was removed from the culture containers when detected. 

## 3. Experimental Procedure

At the start of all experiments, soil moisture was adjusted to 50% of its water-holding capacity. Copper sulphate (CuSO_4_, Sigma, Steinheim, Germany) spiked soils with different concentrations (see Table 2) were prepared for three different experimental setups: avoidance tests, individual placement tests and small-scale gradient tests. For each set of experiments, different spiking solutions were prepared by diluting a specific volume of a copper sulphate stock solution in distilled water (similar final volume for each spiking solution) to attain the desired test concentrations. These spiking solutions were mixed directly in specific portions of soil immediately before its use in experiments. 

### 3.1. Avoidance Tests

Dual chamber avoidance tests were performed with earthworms and springtails following procedures based on the ISO guidelines 17512-1 [21] and 17512-2 [22], respectively. Combinations (control vs. copper spiked soil) using a range of copper contaminated soils (see nominal copper concentrations in Table 2) were tested to define avoidance concentration values (AC_x_) of copper that induce 20, 50 and 80% avoidance behavior (AC_20_, AC_50_ and AC_80_, respectively) for each test species. Additionally, a dual-control test with uncontaminated soil in both sections of the test vessels was performed to validate the test. These tests were performed at 20 ± 2 °C under a photoperiod of 16:8 h, light:dark. 

**Figure 1:**
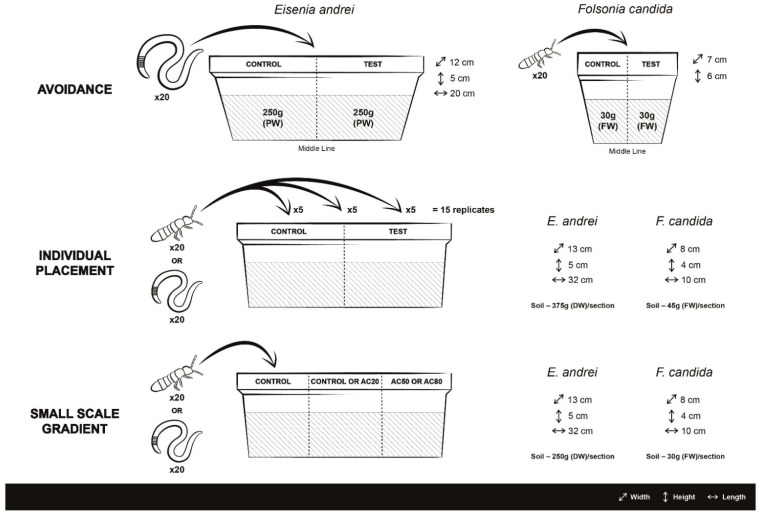
Visual scheme depicting test vessels and experimental procedures for the avoidance, individual placement and small-scale gradient tests.

For avoidance tests with earthworms, each test vessel consisted of a plastic box (20 cm length, 12 cm width, and 5 cm height) divided into two sections by a card divider. In each section, 250 g (dry weight equivalent; DW) of uncontaminated or copper contaminated soil were placed. Twenty adult earthworms were placed in the middle line between both sections after removing the card divider. Five replicates were prepared per combination. For springtails, the procedures adopted were similar to those used for earthworm tests but cylindrical plastic boxes (7 cm diameter, 6 cm height) were used as test vessels and 30 g (fresh weight equivalent; FW) of soil was placed in each section. Twenty springtails 10- to 12-days old (taken from synchronized cultures) were placed in the middle line between sections after removing the card divider. Five replicates were prepared per combination. Samples were obtained from each soil at the start of the experiment to measure initial pH and soil water content. A visual depiction of the test vessels is provided in Figure 1.

For both avoidance tests with earthworms and springtails, the test period was of 48 h, after which plastic dividers were carefully reintroduced separating the two soil test sections. For earthworms, the soil in each section was transferred to plastic trays and the number of earthworms was counted by hand sorting. For collembola, the soil in one section was transferred to a small plastic vessel of the same size. The plastic divider was then removed and the soil in each vessel was flooded with water, a few drops of dark ink were added and after gentle stirring the number of springtails floating on the water surface was determined.

In the following set of experiments, copper concentrations used were defined according to the AC_x_ concentrations estimated for each species in these avoidance tests. These experiments were conducted to empirically demonstrate the avoidance–recolonization hypothesis and validate the RC_x_ concept. For the individual placement and small-scale gradient experiments, slightly larger and different test vessels were used to allow a better division into three sections for the gradient test. As a result, a larger amount of soil (but with the same amount of total soil between individual placement and small-scale gradient tests) was used. The larger test vessels and soil were not used for the avoidance tests as these were conducted following standard guidelines in order to allow a better comparability with other avoidance experiments. The different test vessels and experimental procedures are visually depicted in Figure 1. 

### 3.2. Individual Placement Tests

These assays were adapted from the ISO guidelines 17512-1 [21] and ISO 17512-2 [22] for earthworms and springtails, respectively. The procedures adopted were similar to those used for avoidance tests with some differences. For the individual placement tests with earthworms, plastic boxes (32 cm length, 13 cm width and 5 cm height) were used and each section was filled with 375 g of soil (DW). For tests with springtails, plastic boxes (10 cm length, 8 cm width and 4 cm height) were used and 45 g of soil (FW) was placed in each section. Contaminated soils with copper concentrations corresponding to the AC_20_, AC_50_ and AC_80_ (estimated for each species in the previous set of tests) were tested against the control (uncontaminated) soil. A total of 15 replicates were prepared for each soil combination. These 15 replicates were sub-divided into three groups of 5 replicates where the organisms were introduced in three different locations of the test containers: in the middle line between both sections (as performed in a standard dual chamber avoidance test), in the section with contaminated soil (an avoidance scenario) or in the section with uncontaminated soil (a recolonization scenario). The procedures are visually depicted in Figure 1. In addition to treatments with contaminated soil, ten replicates with uncontaminated soil in both sections were prepared (dual-control test) to demonstrate that no avoidance occurs when the same soil is placed in both sections of the replicates. In this case, in half of the replicates the organisms were placed in the middle line of the test containers and in the other half in one of the sections. After 48 h, the number of individuals in each section was determined adopting the same methodology used in the previous avoidance tests.

### 3.3. Small-Scale Gradient Tests

Each test vessel (same size of those used in the individual placement tests) was divided into three equal sections, using two plastic dividers. For tests with earthworms, 250 g of soil (DW) was placed in each section and for springtails tests, 30 g of soil (FW) was used. Four combinations were tested using uncontaminated soil (CT) and copper spiked soils with corresponding AC_20_, AC_50_ and AC_80_ concentrations for each species. The sequences tested were CT-CT-AC_50_ and CT-CT-AC_80_ (without an intermediate copper contaminated soil) that worked as control for the combinations CT-AC_20_-AC_50_ and CT-AC_20_-AC_80_, respectively. Each combination was performed with 5 replicates for both test species. Soil samples were collected from each treatment at the beginning of the test to measure initial pH and soil water content.

After removing the plastic dividers, 20 individuals of each test species were placed in the border section of the test vessel with uncontaminated soil (Ct). After 48 h, the plastic dividers were reintroduced in their previous positions and the number of surviving organisms was determined in each section following procedures described for the avoidance tests.

## 4. Metal Analysis 

For total copper concentrations, 80–100 mg samples of oven dried (at 105 °C for 12 h) and homogenized soil were used in triplicate for each treatment. Each sample was mixed with 2 mL of 69% nitric acid (PA-AC-ISO, Panreac, Barcelona, Spain) and left under pressure in a PDS-6 system (Loftfields analytical solutions, Neu Eichenberg, Germany) at 150 °C for 10 h. After this period, 8 mL of distilled water was added to the resulting solution and the final volume of 10 mL was transferred to a plastic vial. A blank with no soil was also prepared adopting the same procedure. The accuracy of this analysis was checked using SRM 2709 (San Joaquin soil—standard reference material) certified by the National Institute of Standards and Technology (Department of Commerce, Washington, DC, USA), average recovery of copper was 107.4 ± 15.4% in the reference material.

The extractable copper concentration was determined by stirring 2 g of air-dried soil with 20 mL of 1 M ammonium acetate (CH_3_COONH_4_—Sigma, Steinheim, Germany) solution at 400 rpm for 2 h. After this period the resulting solution was filtered through a Whatman n°1 filter paper disc (Cat. N° 1001150, Maidstone, England) and stored in plastic vials. This procedure was performed in triplicate for each test treatment and a blank with no soil was also prepared. 

Total and extractable copper concentrations were determined by flame AAS (2380 Absorption Atomic Spectrometer, Perkin-Elmer).

## 5. Statistical Analysis

In dual chamber avoidance tests, the significance of the avoidance behavior was tested using the Fisher exact test as described by Natal-da-Luz et al. [23]. The percentage of avoidance response of both *E. andrei* and *F. candida* was calculated according to the formula *A = C − T/N x100* (where *C* is the number of individuals in the control soil, *T* is the number of individuals in contaminated soil and *N* is the total number of individuals [20,21]). The nominal copper concentration at which a specific percentage of avoidance response (AC_x_) is detected was estimated using the PriProbit 1.63 software [33].

Data obtained in the individual placement tests were analyzed by Fisher exact test. This statistical tool was used to compare the distribution of organisms in replicates where collembolans were introduced in the middle line of the test containers (expected distribution) with that of replicates where the organisms were introduced in one of the test sections (observed distribution). The null hypothesis assumes an equal final distribution of the organisms independently of their initial position (either introducing the organisms in the middle line of the test vessels or introducing the organisms in one of the test sections).

In the small-scale gradient tests, data sets were analyzed by Fisher exact tests comparing the proportion of individuals found in the final section (section opposite to that where the organisms were introduced) of the combinations Ct-Ct-AC_50_ and Ct-Ct-AC_80_ (expected distribution) with that of the combinations Ct-AC_20_-AC_50_ and Ct-AC_20_-AC_80_, respectively (observed distribution). In this test, the null hypothesis assumed an equal proportion of colonizing individuals in the final section (AC_50_ or AC_80_ section) in treatments with and without a lower copper concentration (AC_20_) in the previous section.

Fischer exact tests were calculated using the freely available online calculator for contingency tables (available at https://www.graphpad.com/quickcalcs/contingency1/ last accessed 31 January 2022). 

## 6. Results

Chemical and physical characterization of the soil used in all experimental testing is presented in Table 1. To attest for dosing procedures of the test soil, copper concentrations were measured and are presented in Table 2. Total copper concentrations were always higher than nominal concentrations due to the presence of residual copper in the soil. However, both total and extractable copper concentrations measured showed that the relative copper values between test concentrations were consistent with those of nominal concentrations (Table 2).

### 6.1. Avoidance Tests

**Figure 2:**
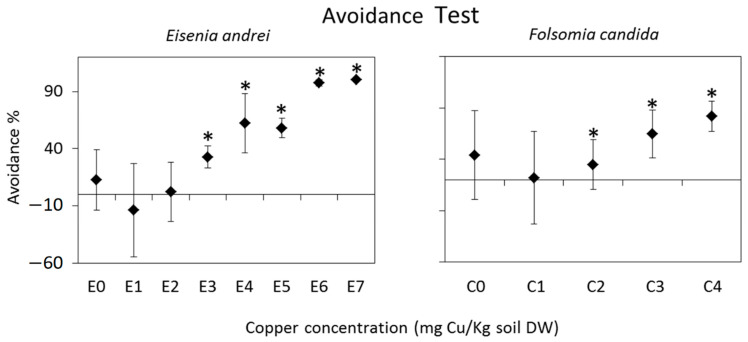
Avoidance tests with *Eisenia andrei* and *Folsomia candida*. Percentage of avoidance (average ± standard deviation; *n* = 5) in test units combining uncontaminated soil with a copper contaminated soil. * indicates significant avoidance response (*p* ≤ 0.05) after Fisher exact test.

Initial avoidance tests were performed to determine avoidance concentration values for all following experimental setups. In these tests mortality was always, on average, lower than 3% in all combinations tested for both test organisms. The avoidance response observed increased with the increasing copper concentrations for both test species (Figure 2). *E. andrei* significantly avoided concentrations higher or equal to 25 mg Cu/kg soil DW, while *F. candida* showed significant avoidance behavior at a nominal copper concentration higher or equal to 200 mg Cu/kg soil DW. The AC_x_ values estimated for both test species are shown in Table 3. 

### 6.2. Individual Placement Test

**Figure 3:**
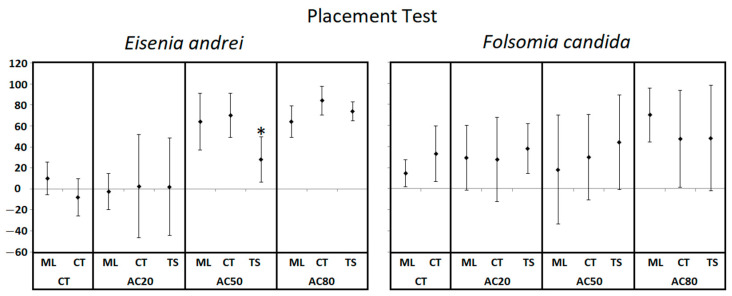
Placement tests with *Eisenia andrei* and *Folsomia candida*. Percentage of avoidance (average ± standard deviation; *n* = 5) in test units combining uncontaminated soil (Ct) with Ct or copper contaminated soils equivalent to AC_20_, AC_50_ and AC_80_ concentrations. For each of these combinations individuals were placed in the middle line (ML), in the control soil (CT), or in the test soil (TS). * indicates a significant difference from the combinations where individuals were placed in the middle (*p* ≤ 0.05) after Fisher exact test.

Individual placement tests aimed at demonstrating that the final distribution of organisms after 48 h is not dependent on their initial position (middle line, control/test soil) in dual chamber avoidance tests combining different copper concentrations (AC_20_, AC_50_ and AC_80_) with uncontaminated soil (Figure 3). In these experiments, no mortality was observed for *E. andrei* and mortality of *F. candida* was, on average, lower or equal to 6% for all treatments. Regarding the statistical analysis of the different treatments (placement in different sections vs. placement in the middle line) no significant difference was found except for *E. andrei* in the AC_50_ treatment where the combination with individuals placed in the test soil were significantly different from controls (*p* < 0.05, introduction of organisms in middle line). 

### 6.3. Small-Scale Gradient Tests

**Figure 4:**
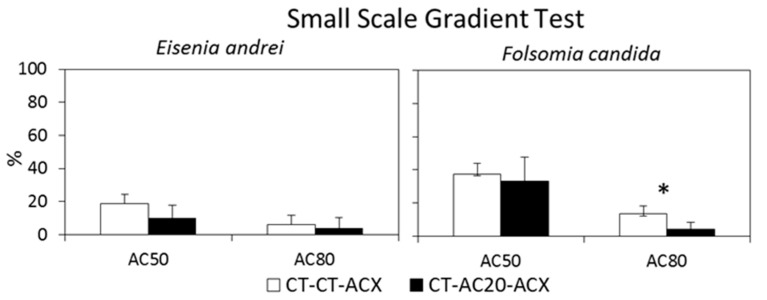
Small-scale gradient tests with *Eisenia andrei* and *Folsomia candida*. Percentage of individuals (average ± standard deviation; *n* = 5) in the section with the highest copper contaminated soil in three compartment test units combining uncontaminated soil (CT) with copper contaminated soils (AC_20_, AC_50_ or AC_80_) using as intermediate concentrations CT (white bars) or AC_20_ (black bars) concentrations. * indicates a significant difference between combinations with and without an AC_20_ intermediate concentration (*p* ≤ 0.05) after Fisher exact test.

This experimental setup was performed to demonstrate that lower concentrations (AC_20_) in a contamination gradient do not influence the number of organisms reaching a higher concentration (AC_50_/AC_80_). In these experiments, two combinations were used (CT–AC_20_–AC_50_ and CT–AC_20_–AC_80_) which were tested against the respective controls with no intermediate concentration (CT–CT–AC_50_ and CT–CT–AC_80_). The percentage of individuals found in the last section (AC_50_ or AC_80_) of the three compartment test units, for both the control (CT-CT-AC_x_) and test (CT-AC_20_-AC_x_) combinations is presented in Figure 4. 

In these experiments, no mortality was observed for *E. andrei* and the mortality of *F. candida* was, on average, lower than 5% in all combinations. No significant differences between the control (CT-CT-AC_x_) and test (CT-AC_20_-AC_x_) distributions in combinations with the same AC_x_ were found for *E. andrei*. For *F. candida*, a significant difference between the control and test distributions was found only for combinations with AC_80_ (*p* = 0.02; Figure 3). 

## 7. Discussion

The avoidance AC_50_ values obtained for *E. andrei* (49.5 mg Cu/kg DW) were lower than the previously reported AC_50_ values of 181.1 mg/kg and 94.08 mg/kg, in Lufa 2.2 [34] and OECD artificial soil [35], respectively, but were much higher than the AC_50_ value of 1.7 mg/kg reported by Greenslade and Vaughan [36], also in OECD artificial soil. All reported studies followed similar procedures and differences in sensitivity would most likely be due to other factors such as differences in soil properties, the source of copper used for spiking or the test species used. 

For soil properties, it is known that properties such as pH, organic carbon content, clay content and cation-exchange capacity influence the toxicity of copper in chronic assays [37], but the extent of their contribution especially for behavioral endpoints is still not fully understood. In fact, while soil properties such as pH are good predictors of metal solubility, it is still not possible to infer toxicity based on metal solubility [38]. 

In addition to soil properties, the source of copper used for soil spiking (i.e., copper sulphate, copper nitrate, copper oxide, etc.) could affect the sensitivity of organisms by affecting copper bioavailability in soil [39]. Copper form, could in part explain the large difference in sensitivity observed in both studies using OECD soil [35,36] but unfortunately, one of the studies conducted in OECD soil did not report the source of copper used for spiking [36]. For the remaining studies, one used copper nitrate [35] and both our study and the study with Lufa 2.2 soil [34] used copper sulfate. 

Finally, unlike the remaining studies, which were conducted with *E. andrei*, the study by Xing et al. [35] used the species *E. fetida* which, despite having comparable sensitivity, could, in conjunction with the other factors, also contribute to some of the differences observed. Further information and examinations would be required to understand these differences in sensitivity especially for both the studies conducted in OECD soil.

Regarding *F. candida*, the AC_50_ values obtained (1007.71 mg/kg) were much higher than those reported in the literature (61.2 mg/kg [36] and 18 and 17 mg/kg [40]). This difference could be due to differences in copper form used for spiking (not reported in either studies) and soil properties (for Greenslade and Vaughn [36]) which as explained above, can affect copper toxicity to soil organisms. Additionally, for the study conducted by Boiteau et al. [40], differences could be due to the methodology applied, where unlike the remaining studies conducted in soil, the avoidance AC_50_ was only determined using a Petri dish method. 

In the individual placement tests, it was demonstrated that the position in which earthworms and collembolans are placed in a dual chamber avoidance test does not influence their final distribution. Only in one treatment was there a significant difference observed, for *E. andrei* when individuals were placed in the test soil at the AC_50_ level. Thus, overall, the standard procedure used in avoidance tests (placing individuals in the middle line) provides the same response as if a recolonization scenario (placing the individuals in the control soil) or avoidance scenario (placing individuals in contaminated soil) is considered. This empirically demonstrates the avoidance–recolonization hypothesis, i.e., individuals which do no avoid a certain soil are able to colonize it (AC_x_ = RC_(100−x)_). Results obtained corroborate those found by Heupel [41] that demonstrated that in dual chamber avoidance tests placing individuals in the control or in a soil spiked with the fungicide Betanal, the same final distribution is observed for several collembolan species (including *F. candida*). 

Regarding the influence of lower concentrations in a contamination gradient, the overall results experimentally demonstrate that intermediate copper concentrations did not influence the dispersal of colonizers to higher copper concentrations in a contamination gradient except for one combination for *F. candida* (borderline *p* value of 0.02). For *F. candida,* the influence of an intermediate concentration detected could be due to a factor of time and scaling. Further research exploring larger temporal incubation periods would be interesting to check how time influences the dispersal and avoidance behavior of organisms when exposed to a gradient of contamination. 

Overall, the results obtained support that individuals which are not sensitive enough to avoid a higher concentration of contaminant are also equally insensitive to lower concentrations of contaminants. As such, the expected avoidance response of the test organisms to a certain copper concentration is not affected by its previous exposure to lower concentrations. This is an important demonstration for the avoidance–recolonization hypothesis in that modelling the recovery potential of the most contaminated patches is not dependent on the surrounding patches with lower concentrations. This is particularly important for their application in risk assessment schemes where the full extent of the contamination patchiness is not always known. 

In the scientific literature, dispersal and avoidance behavior for metal contaminated gradients, has been previously considered. Bengtsson et al. [42] for instance found that modelled distributions in a gradient of metal contamination, presented the closest fit to observed distributions when individuals were able to detect differences between metal concentrations, as they moved between soils and remained in the less contaminated ones. Additionally, Meli et al. [19], found when modelling the spatial distribution of *F. candida* in simulated copper contaminated patches, that avoidance behavior acts as a key mechanism allowing for non-contaminated patches to act as population reservoirs and donor areas in heterogeneous contamination scenarios. This originates a decrease in extinction probability of the population when compared to scenarios with a spatially homogeneous contamination, and a more realistic assessment of the risk for the species. The shift in the distribution of organisms due to contamination was also demonstrated in a microcosm experiment with patchily contaminated soil that found that microarthropods avoided the more contaminated soil patches which had lower abundances than clean soil patches [26]. 

The current study as well as previous research have highlighted the importance and potential of measuring avoidance response when predicting/modelling the recolonization of contaminated habitats and, ultimately, ecological recovery. Specifically, the avoidance–recolonization concept can allow for existing avoidance data to be selected and implemented in modeling approaches. 

In fact, the avoidance–recolonization hypothesis demonstrated here for soil with the model species, *F. candida* and *E. andrei* (individual placement and small-scale gradient experiments), has also previously been demonstrated in the aquatic compartment using the model species *Daphnia magna* [28]. In the aquatic avoidance study, placing *D. magna* in the least contaminated compartment or a uniform distribution along different compartments of a contamination gradient produced a similar distribution and AC_50_ and RC_50_ values were not significantly different. 

In demonstrating the avoidance–recolonization concept, it inherently demonstrates that the recolonization of a contaminated soil is a density-dependent process modulated by organism dispersal and contaminant concentrations over time. However, further research is still required and must be carefully considered when looking to validate the approach and explore predictions at larger temporal and spatial scales. 

From the contaminant perspective, it is important to consider the fate of the contaminant in soil but also its mechanisms of action, which can affect avoidance behavior dynamics and consequently recolonization processes. For instance, dimethoate was found to impair the locomotion of *F. candida* providing false negative avoidances [43] and etofenprox (under the formulation Trebon 30 EC) was found to promote locomotory hyperactivity followed by very reduced locomotory activity [44]. 

In addition to contaminant mode of action, the interaction between time, population density and resource availability will play an important role at larger and more realistic scales. Specifically, there could be a cost benefit response to consider where organisms will only look to recolonize surrounding patches if habitat or resource conditions are more favorable. The role of time on avoidance behavior has been previously explored and did not appear to play a significant role, but the timescales considered were up to a maximum of 7 days while for more realistic timescales (i.e., months) these dynamics might change [45]. 

Finally, the ultimate goal for risk assessment of contaminated soils is to understand the role of avoidance behavior on the recovery and recolonization processes for natural communities. In this sense, the current avoidance–recolonization concept provides an important first step and conceptual framework for modelling approaches to understand the role of avoidance behavior in recolonization processes but is limited to the available data for the currently used model species. In order to understand and extrapolate this information for natural communities, will require a good understanding of their ecological traits (i.e., dispersal ability), how species interactions affect avoidance behavior dynamics and that either, currently tested species in avoidance tests are adequately representative of sensitivities in natural communities or a better understanding of their chemical sensitivity.

## 8. Conclusions

Overall, while further research is necessary, this study demonstrated the avoidance–recolonization hypothesis by evidencing that organisms which avoid a soil cannot recolonize that same soil (AC_x_ = RC_(100−x)_). As such, standard procedures provide the same response as if a recolonization or an avoidance scenario is considered. Additionally, it was demonstrated that an organism’s recolonization of a contaminated soil patch is not affected by lower intermediate patches along a gradient of contamination. As a result, avoidance concentrations could be the tool needed to help understand the recolonization potential of a contaminated soil in spatial modelling approaches. While this concept only implies a paradigm shift (from avoidance to recolonization), its application for modelling tools is extremely valuable. In fact, in spatially based population models if contaminant concentrations, dispersal ability and sensitivity of organisms are well known, it should be possible to predict the necessary “time to recovery” at realistic spatial scales when considering both internal and external recovery processes. 

## Figures and Tables

**Table 1:** Chemical and physical characterization of the test soil.

Parameter	Value
Water-holding capacity	36.2 ± 0.4%
Organic matter	3.3 ± 0.1%
pH (KCl 1 M)	6.9
Total N	0.83 mg/g
Cation-exchange capacity	0.0125 cmol/g
Sand	62.40%
Silt	21.20%
Clay	16.40%
Soil texture class	Sandy-loam
Soil type	Cambisol

**Table 2:** Nominal concentrations (expected) and total and extractable concentrations (measured; average ± standard deviation) of copper in soil treatments used in the avoidance tests, individual placement tests, small-scale gradient tests and large-scale gradient tests, using *Eisenia andrei* or *Folsomia candida* as test organisms. E0-E7 and C0-C4 refer to treatments used in avoidance tests with *E. andrei* and *F. candida*, AC_20_-AC_80_, avoidance concentrations (estimated at the initial avoidance tests), used in the other tests for each species, respectively.

		Nominal (mg/kg)	*n*	Total Copper	Extractable Copper
				(mg/kg)	(mg/kg)
*Eisenia andrei*	E0/CT	0	15	29.37 ± 2.64	0.06 ± 0.12
	E1	5	3	34.45 ± 3.77	0.05 ± 0.03
	E2	10	3	48.25 ± 3.59	0.06 ± 0.1
	E3	25	3	54.27 ± 7.49	0.58 ± 0.23
	E4	50	3	89.12 ± 5.42	1.20 ± 0.07
	E5	100	3	140.60 ± 3.71	5.42 ± 0.45
	E6	200	3	239.99 ± 31.36	22.11 ± 2.60
	E7	400	3	551.57 ± 30.6	73.2 ± 5.80
	AC_20_	20	9	50.79 ± 4.48	0.42 ± 0.15
	AC_50_	50	6	74.49 ± 9.59	1.90 ± 0.28
	AC_80_	100	6	147.09 ± 15.43	5.66 ± 0.61
*Folsomia candida*	C0/CT	0	9	30.21 ± 3.77	0 ± 0.16
	C1	100	3	185.29 ± 18.44	6.87 ± 0.44
	C2	200	3	330.84 ± 21.46	26.15 ± 0.27
	C3	800	3	1173.89 ± 22.30	159.25 ± 21.66
	C4	1600	3	2257.49 ± 34.95	709.91 ± 77.53
	AC_20_	300	6	382.36 ± 47.44	42.50 ± 9.80
	AC_50_	1000	6	1061.51 ± 71.79	264.28 ± 46.70
	AC_80_	3300	6	3765.78 ± 276.73	1466.79 ± 143.88

**Table 3:** Avoidance concentrations (AC_x_; with corresponding 95% confidence intervals) estimated for *Eisenia andrei* and *Folsomia candida* exposed to concentration gradients of copper spiked soil. AC_x_ values are expressed in mg of Cu per kg of soil (dry weight equivalent).

AC_x_	*Eisenia andrei*	*Folsomia candida*
AC_20_	21.8 (3.9–39.7)	301 (49.8–527)
AC_50_	49.5 (20.6–78.0)	1008 (601–1890)
AC_80_	112 (70.9–237)	3371 (1824–26,987)

## Data Availability

Data are available as Supplementary Material.

## Supplementary Materials

The following supporting information can be downloaded at: https://www.mdpi.com/article/10.3390/toxics10030127/s1. The data for metal concentrations and avoidance experiments are provided in the supplementary data (DataRC.xlx).
